# Thermodynamics of Intrinsic Reaction Coordinate (IRC) Chemical Reaction Pathways

**DOI:** 10.3390/e27040390

**Published:** 2025-04-07

**Authors:** Frank Weinhold

**Affiliations:** Theoretical Chemistry Institute and Department of Chemistry, University of Wisconsin-Madison, Madison, WI 53706, USA; weinhold@chem.wisc.edu

**Keywords:** thermodynamics, intrinsic reaction coordinate, natural resonance theory, quantum cluster equilibrium

## Abstract

We address the scientific “time” concept in the context of more general relaxation processes toward the *Wärmetod* of thermodynamic equilibrium. More specifically, we sketch a construction of a conceptual ladder of chemical reaction steps that can rigorously bridge a description from the microscopic domain of molecular quantum chemistry to the macroscopic materials domain of Gibbsian thermodynamics. This conceptual reformulation follows the pioneering work of Kenichi Fukui (Nobel 1981) in rigorously formulating the *intrinsic reaction coordinate* (IRC) pathway for controlled description of non-equilibrium passages between reactant and product equilibrium states of an overall material transformation. Elementary *chemical reaction steps* are thereby identified as the logical building-blocks of an integrated mathematical framework that seamlessly spans the gulf between classical (pre-1925) and quantal (post-1925) scientific conceptions and encompasses both static and dynamic aspects of material change. All modern chemical reaction rate studies build on the apparent infallibility of quantum-chemical solutions of Schrödinger’s wave equation and its Dirac-type relativistic corrections. This infallibility may now be properly accepted as an added“inductive law” of Gibbsian chemical thermodynamics, the only component of 19th-century physics that passed *intact* through the revolutionary quantum upheavals of 1925.

## 1. Introduction

*What then is time? If no one asks me, I know what it is. If I wish to explain what it is to him who asks me, I do not know.*

—St. Augustine (Fifth Century).

The kind invitation to contribute to this Special Issue of *Entropy* gives opportunity to interweave related strands of the author’s personal scientific interests over the decades since their intersection with the original finite-time thermodynamics (FTT) studies of the 1970s [1]. The discussed topics include the original 1975–1976 papers on thermodynamic geometry [2,3,4], analytic extensions of frequency-dependent polarizabilities to the complex plane of imaginary-frequency values [5], as well as related methods for obtaining the lifetime of unstable species as the imaginary component of system energy eigenvalue after complex-coordinate rotation of the Schrödinger Hamiltonian operator [6]. In each case, these studies touch upon Einstein’s extensions of Euclidean geometry into the large-scale Riemannian structure of general relativity and 3*d*-spatial coordinates into the 4*d* space–time coordinates of special relativity, spanning microscopic and macroscopic domains of physical and chemical description.

The identification of the measurable lifetime of a metastable species with the imaginary component of an analytically continued solution of Schrödinger’s equation naturally draws attention to the parallels with other physical descriptors of spontaneous temporal change. These include the *entropy* property, whose monotone *increase* in spontaneous changes of isolated systems underlies the second law of thermodynamics. These parallels also hint at fundamental connections between time-like measures of spontaneous change as perceived in microscopic and macroscopic domains of chemical reaction dynamics, the focus of present study.

In the present work, we suggest how to build formal bridges that can better connect the logic, mathematical formalisms, and conceptual perspectives of the microscopic (Schrödinger/Dirac/Pauling) domain of atoms and molecules with the macroscopic (Gibbs/Boltzmann) domain of bulk material properties in equilibrium or non-equilibrium states. In each case, our viewpoint is that of a *chemical* practitioner, in recognition of chemistry’s role as the “central science” [7] that bridges many aspects of marine, geological, biological, atmospheric, and interstellar studies in the natural sciences.

Of particular interest are the optimal mathematical and conceptual building-blocks of a unified theoretical framework that can smoothly bridge the ca. 10^23^-fold transition from microscopic to macroscopic distance and energy scales. Such a framework necessarily deals with *change* (*t*-dependence) as well as the time-independent *Wärmetod* of the terminal equilibrium-state limit. It must also grapple with the *logical* gap that separates the perceptions of classical (pre-1925) scientists from those following discovery of the quantal Schrödinger/Dirac equations (and equivalent matrix-algebraic formulations) in 1925–1928 [8]. At a still deeper level, it must strive to reconcile contending preferences for inductive (axiom-free) vs. deductive (axiom-based) modes of scientific reasoning. In each case, bridge-building begins from one side or the other of a conceptual divide. In the following, we deal with these divides in approximate chronological order.

## 2. Inductive Scientific Tenets and Their Survival in the Face of Conceptual Revolution

We begin (as does every newborn child) with a rigorously inductive (axiom-free) approach to describing the nature that surrounds us. What are gradually perceived as experiential *patterns* or regularities of past natural events become the basis for increasingly confident prediction of future events. Any violation of such inductive expectation is cause for its dismissal from consideration as a possible law of nature, and only those regularities that remain strictly exception-*free* to all competent observers are considered proper starting points for rigorous deductions of other consequences. In this manner, one gains consensus for the universality of conclusions from laws that are strictly self-consistent with direct observations from nature.

Uniquely among the natural sciences, *equilibrium chemical thermodynamics* epitomizes such inductive foundations. As masterfully formulated by J. W. Gibbs [9], the first and second laws of thermodynamics that govern the thermal behavior of all chemical materials can be rigorously expressed in terms of low-order differential relationships among the small set of measurable properties [*U* (internal energy), *S* (namesake of this journal), *V* (volume), and *N*_i_ (mole number of the *i*th pure substance)] that fully specify the *equilibrium state* of the macroscopic chemical system. As Gibbs recognized, the two alternative but fundamentally equivalent representations of thermodynamic relationships (involving 1st- and 2nd-order derivatives only) can be based solely on the following energy-based (1a) or entropy-based (1b) functional expressions:

*U* = *U*(*S*,*V*,{*N*_i_}) (1a)

*S* = *S*(*U*,*V*,{*N*_i_}) (1b)

whereas the (*nearly* equivalent) Legendre transformations of various types [10] require subsidiary assumptions of higher-order differentiability and lower generality.

Also uniquely among the natural sciences, Gibbsian equilibrium thermodynamics passed *seamlessly* through the quantum revolutions of 1925 without need for an iota of revision. If anything, the discovery of bizarre quantal phenomena that defied classical description (including Heisenberg’s uncertainty principle and associated non-vanishing vibrational fluctuations that persist even at *T* = 0 K) only *solidified* Gibbs’s conclusions concerning unwarranted third-law and higher-order differentiability assumptions that were entertained by others. These considerations suggest that building on thermodynamic-like inductive notions is a *robust* foundation for a properly unified description of electronic interactions from microscopic to macroscopic limits.

Consistent with all post-1925 measurements of physical and chemical properties, one can say without exception that *all* such critical measurements, carried out to the error limits of current technology, are found to be in *exact* compliance with corresponding solutions of the quantum-mechanical Schrödinger equation and its Dirac-type corrections. This conclusion applies not only to the small number of cases for which exact analytic solutions of Schrödinger’s equation are known, but more importantly to the numerical solutions determined by systematically improvable algorithms and currently available computational resources, including rigorous upper- and/or lower-bound error limits [11].

Therefore, until proven otherwise, one can take the *truth of the Schrödinger equation* (and its proper relativistic generalization) as a veritable inductive law of chemical thermodynamics. This allows state-of-the-art quantum chemistry methods to acquire axiomatic-like status in deducing authentic patterns of chemical behavior across the broad span between microscopic (electronic) vs. macroscopic (thermodynamic) levels of description.

Currently, the broad suite of modern density functional theoretic (DFT) methods, based on fundamental contributions of Walter Kohn (Nobel 1998) and others [12], are the practical quantum chemistry tools of choice for realistic approximations to authentic solutions of Schrödinger’s equation in broad areas of chemical research. DFT-based approaches are based on rigorous theorems for the adequacy of correct *electron density* [ρ(**r**)] description to provide exact variational solution of Schrödinger’s equation.

This in turn calls conceptual attention to the electronic *orbitals* {φ_i_(**r**)} and non-negative *populations* {*n*_i_} (compliant with the Pauli exclusion principle) that quite generally allow the expression of the electron density as a *convex* combination of squared-orbital densities,

ρ(**r**) **=**
*n*_1_|φ_i_(**r**)|^2^ + *n*_2_|φ_2_(**r**)|^2^ + … ≥ 0 (2a)

with orbital occupancies summing to the total number of electrons (*N*),

∑_i_ *n*_i_ = *N* (2b)

For conceptual purposes, optimal orthonormal orbitals are frequently chosen to emulate the 1-center (lone-pair) and 2-center (bond) electron-pairs of familiar localized Lewis-structural bonding patterns that long pre-date the discovery of quantum mechanics, but this choice [13] is a matter of convenience. Mathematically optimal population analysis [14] of such DFT or higher-level density distributions in terms of localized atomic orbitals is now a routine by-product of electronic-structure calculations conducted by chemists in every area of specialization. All such efforts are supported by inductive faith in the accuracy of current methods for obtaining systematically improvable solutions of Schrödinger’s equation that lead reliably to agreement between theory and measurement (within respective error limits) in all known cases.

## 3. Quantum Cluster Equilibrium Theory of Fluid Properties

Still another bridge between the Schrödinger-based description of molecular species and the thermodynamic-level description of the associated fluid phase behavior is provided by the *quantum cluster equilibrium* (QCE) model of molecular cluster interactions [15]. QCE methodology was initially developed for simple phase-transition properties of pure fluids but subsequently extended by Kirchner and coworkers to binary mixtures and more complex solution properties [16].

As the name implies, QCE methodology employs the quantum chemistry of molecular *clusters* (of pure or binary-mixed types) in (*T*,*P*)-dependent equilibrium mixtures to predict the properties of the associated macroscopic fluid-phase diagram. In the case of water, the vapor-phase region of the equilibrium QCE distribution is dominated (as expected) by H_2_O monomers, while the liquid-phase distribution is dominated by *ring*-like clusters of 5- and 6-member topology [17], as significantly favored by strongly *cooperative* (*non*-pairwise-additive) aspects of hydrogen bonding. Inclusion of sparsely populated *ionic* water clusters in the equilibrium QCE mixture also allows one to also predict (*T*,*P*)-dependent *pH* values for the aqueous fluid phase [18]. With inclusion of clathrate-like bucky-ball clusters, one also finds formation of metastable *solid* phases that connect to the fluid phases in characteristic triple-point fashion [19]. All these (systematically improvable) features of the QCE model partition function are qualitatively or semi-quantitatively consistent with known experimental properties of the macroscopic phase diagram. The QCE model can therefore be seen as an ever-improvable formal “bridge” between supramolecular clusters of the quantal regime and the macroscopic phases of classical Gibbsian thermodynamics.

## 4. Non-Equilibrium Thermodynamics on IRC Reaction Pathways: An Integrated Resonance-Mechanistic View

Far the greatest challenge to further the extension of Gibbsian-like inductive conceptions is to find a corresponding bridge that spans the full range of equilibrium (static; *t*-independent) states and non-equilibrium (dynamic; *t*-dependent) phenomena. As conceptual building-blocks for such non-equilibrium transformations that intrinsically involve time-like variability, we employ Fukui’s *intrinsic reaction coordinate* (IRC) [20] concept for each elementary chemical reaction underlying the material change. The IRC coordinate allows the entire pathway of an elementary chemical reaction (from equilibrium reactant- to final product complex) to be *uniquely* determined from the stationary *transition*-state geometry that lies at the highest energy along the IRC pathway. In this manner, complex material changes can be systematically broken down into IRC-based pathways through the non-equilibrium regions that connect each equilibrium reactant and product species of the overall process.

Note that the practical application of the IRC concept to describe each non-equilibrium transformation (bond-rearranging, configurational, torsional, …) of a general chemical process relies only on standard quantum-chemical methodology and requires *no* invocation of an empirically constructed *t*-dependent “molecular dynamics” or control operator to describe the chemical reaction process. IRC-based description of chemical relaxation events thereby differs in fundamental respects from dissections into perturbatively small deviations from ideal adiabaticity such as assumed, e.g., in Kosloff’s quantum thermodynamics viewpoint [21], which aims to “insert” quantum dynamics into thermodynamics, even though Gibbsian thermodynamics remains *identical* to its origins that long preceded the 1925 quantal revolution.

By dissecting the overall macroscopic process into the underlying IRC-based chemical reactions in this manner, we gain equilibrium footholds at the terminal *states* of reactant and product species in each non-equilibrium reactive step, which thereby form a connected ladder of reactive-type events that comprehensively compose the perceived overall material change. Well-defined properties of the macroscopic process can thereby be based on measurable *changes of state* between the microscopic reactant and product species for each underlying step of the composite manifold of IRC pathways. As remarked above, modern electronic structure programs routinely provide the (*T*,*P*)-dependent enthalpic and free-energy values of chosen reactant and product chemical species of each step, allowing direct comparison with thermodynamic measurements on the corresponding macroscopic process under chosen temperature and pressure conditions.

Still closer connections can then be drawn between the Schrödinger-based descriptors of a chemical reaction and the measurable progress variables of the corresponding macroscopic process. In the Born–Oppenheimer approximation (which generally suffices for practical chemical purposes), Pauling’s resonance-theoretic concepts for describing the electronic delocalization of individual molecular species can be readily extended to *natural resonance theory* (NRT) analysis [22] of individual points along a chosen pathway on the chosen *E*({***R*_A_**}) potential energy surface (PES). Similar NRT evaluations could also be performed along the extremal-type PES pathway that underlies the original FTT methodology of Andresen and coworkers. As in Pauling’s original heuristic formulation of resonance theory, NRT analysis expresses the delocalized electron-density distribution ρ(**r**) as a convex combination

ρ(**r**) = ∑_α_ *w*_α_ ρ_α_(**r**) (3a)

of those for possible Lewis-like bonding patterns {ρ_α_(**r**)}, with corresponding resonance weightings {*w*_α_} satisfying

1 ≥ *w*_α_ ≥ 0, all α (3b)

and summing to unity

1 = ∑_α_ *w*_α_ (3c)

If *b*_AB_^(α)^ denotes the integer number of bonds between atoms A and B in the Lewis-like bonding pattern for electron-density contribution ρ_α_(**r**), the overall *natural bond order* (*b*_AB_) for each interatomic A and B pair is evaluated as

*b*_AB_**=** ∑_α_ *w*_α_ *b*_AB_^(α)^ (3d)

in accordance with Pauling’s original resonance precepts. Note, however, that the NRT definition (3d) extends seamlessly to *all* atoms of the chosen system, whether considered to be members of the same molecule or not.

Similar to Equations (2a) and (2b) above, the resonance-theoretic Equations (3a)–(3d) thereby identify each such resonance-hybrid electronic distribution along a reactive pathway as a “resonance hybrid” of those for contributing Lewis-like bonding patterns. This in turn allows the resonance weightings {*w*_α_} to be understood as population-type *percentage*s of total electron density at the chosen point of the PES, corresponding to the “charge transfer” terminology that is often employed to describe resonance-type electronic delocalization. Further mathematical and computational details of NRT algorithms [22] are described elsewhere.

The resonance-theoretic expressions seem to provide the most general mechanistic representation of chemical transformation from one bonding pattern to another. All such quantal descriptors are in sharp contrast to the classical-type dipole–dipole rationalizations that were often invoked for such intrinsically stabilizing resonance-type phenomena. The convexity properties (3b) and (3c) of NRT weightings may also be contrasted with the more familiar LCAO-MO-type orbital mixings (involving linear combinations of complex coefficients of opposite signs and arbitrary magnitudes) that are often invoked from the unitarity property of electronic wavefunction theory.

## 5. Illustrative Example: Nitrosamine Degradation to Diazonium Ion

**Figure 1:**
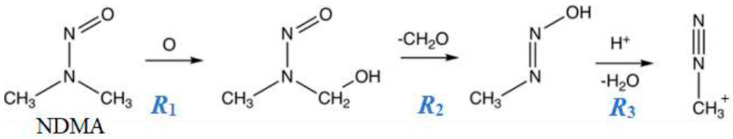
Schematic mechanism of metabolic activation of nitrosamine (NDMA) to diazonium methylating agent in three formal reaction steps ***R*_1_**, ***R*_2_**, and ***R*_3_** (adapted from *Wikipedia* for “nitrosamine”).

As a rather randomly chosen example, we illustrate the IRC-based “NRT portrait” of an elementary chemical transformation pertaining to carcinogenic properties of nitrosamines. Figure 1 depicts the sequence of reactions ***R*_1_**–***R*_3_** by which N-nitrosodimethylamine (NDMA) is converted to a nitrosonium cation that can methylate cytosine and obstruct its proper pairing with guanine in DNA. Reaction ***R*_1_** represents the schematic replacement of H by OH at the methyl site, and ***R*_3_** is similar to *E*1-elimination mechanism for acid-catalyzed ethanol dehydration. Here, we focus attention on the central ***R*_2_** (formaldehyde-elimination) step of diazonium conversion.

**Figure 2:**
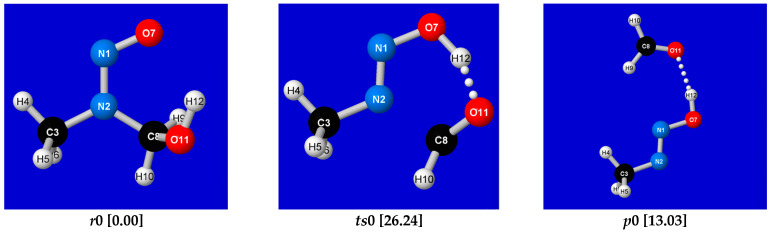
Optimized ground-state-reactant (*r*0; **left**), transition-state (*ts*0; **center**), and product (*p*0; **right**) geometries and relative energies (kcal/mol) for ***R*_2_** reaction step of NDMA conversion to mutagenic methyldiazonium cation with expulsion of formaldehyde.

**Figure 3:**
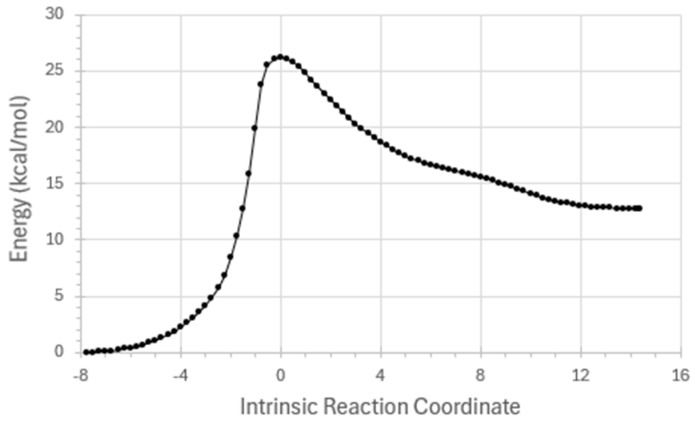
Calculated *E*(*IRC*) energetic variations along the IRC pathway for ***R*_2_** reaction step of NDMA → diazonium conversion.

**Figure 4:**
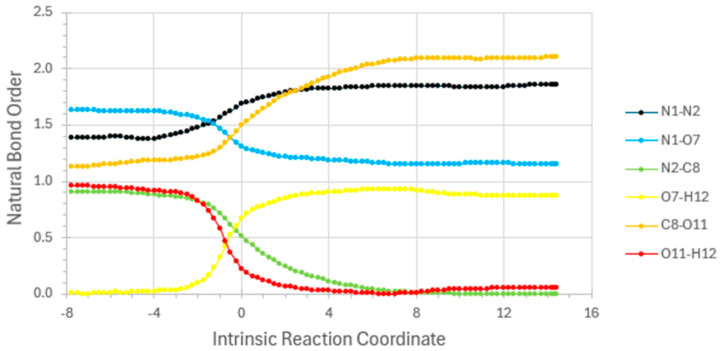
NRT bond-order variations along the IRC pathway for ***R*_2_** reaction step.

Simple DFT-level description of reaction step ***R*_2_** is summarized in Figure 2, Figure 3 and Figure 4. Figure 2 displays ground-state-reactant (*r*0), transition-state (*ts*0), and product (*p*0) geometries and relative energies for the ***R*_2_** reaction step, as calculated at a common DFT level (B3LYP/6-311++G**/D3). Figure 3 displays the full *E*(*IRC*) energy profile, and Figure 4 displays the corresponding variations of NRT bond orders {*b*_AB_(*IRC*)} along the IRC reaction pathway. [Sample *Gaussian-16* input (.gau) and output files for calculating the energy and NRT bond orders for individual points on the IRC are available from the author upon request. Many details of the tandem *G16/NBO7* programs that perform these calculations—now commonly hosted in chemistry research centers but available also in laptop configurations—are described in the respective *Gaussian* or *NBO* websites.]

The interatomic curves in Figure 4 represent the formation [*b*_O(7)H(11)_, yellow] or dissociation [*b*_O(11)H(12)_, red; *b*_N(2)C(8)_, green] of bonds to the main CH_3_NNO substrate, as well as the principal resonance bond-shifts [*b*_C(8)O(11)_, gold; *b*_N(1)N(2)_ black; *b*_N(1)O(7)_, blue] within the substrate. Note that the *sub*-integer NRT bond orders for “inter”molecular interactions differ in *no* significant respect from conventional Pauling-type *supra*-integer bond orders for “intra” molecular interactions. The former correspond to the extension of resonance conceptions into the “no-bond” domain of H-bonding and related X-ogen interactions, which Pauling stoutly opposed until late in his career [23].

Note that the IRC coordinate of the horizontal axis in Figure 3 is indeed a valid “time-like” measure of progress from one stationary state of reaction to another. This follows from the fact that the vertical *E*(*IRC*) energy curve as plotted in Figure 3 conforms to the idealized reaction-energy profile that underlies rigorous transition-state theory of chemical kinetics [24] as employed in every modern laboratory study of molecular reaction dynamics. The “lifetime” associated with the metastable transition-state feature at *IRC =* 0 might be separately evaluated with the complex-coordinate rotation methodology of Ref. [6]. But more to the present point, every NRT bond-order value as displayed in the vertical axis of Figure 4 is found to satisfy the expected [25] strong bond order-bond length (BOBL) and related correlations that can successfully predict a variety of MW/IR/UV/NMR properties that are measurable over the course of chemical reaction. For example, if spectroscopic monitoring establishes that the point of *equal* O(7)H(12) and O(11)H(12) signal [“*half*-transfer of H(12) from O(7) to O(11)”] occurred Δ*t* UTC clock-ticks prior to the highest-energy point (*IRC* = 0) of the reactive sequence, one can affix Δ*t* as the “standard-*t*” value at the corresponding red/yellow curve-crossing near *IRC* ≈ –1.0 in Figure 4.

Note also that the IRC pathway as defined by Fukui [20] has no “near-adiabatic” character nor other resemblance to the pathway envisioned by Kosloff, as can be verified from calculated values of energy, enthalpy, and Gibbs free energy that are included in *Gaussian* output for each step of the IRC search. However, the *intrinsic* property of the IRC insures optimal utility for practical applications throughout the modern chemical and biochemical research domain. As found in many examples—including those for configurational, torsional, or enantiomeric isomerism and long-range supramolecular aggregation—the IRC displays the essential *continuity* of cooperatively-coupled resonance-type interactions throughout the intra- and intermolecular domain in a chemically intuitive manner. Further details of NRT-based mechanistic description follow the pedagogical logic of previously published examples [26].

## 6. Mathematical and Physical Logic: Seeking Unity

The preceding discussion points to what may be perceived as a fundamental logical dichotomy between mathematical and physical sciences. The mathematical sciences epitomize the realm of *deductive* logic, where a chosen set of axioms (not to be questioned further) lead by the strict rules of deductive logic to an entire subject-area of mathematical relationships. Many such mathematical niches are valued for their near-magical connections to physical phenomena, but others are not, described instead as pure mathematics not to be tinged with associations to practical applications.

The physical sciences have frequently benefited from finding their close associations with one or more established fields of mathematical theory. However, each area of physical research is intrinsically constrained by facts of nature as obtained through the specialized research methodologies of its experimental practitioners. If experimental facts are found to conflict with an adopted mathematical framework, that framework must be abandoned or revised until satisfactory agreement between mathematical assumptions and experimental reality is recovered. As one example, the mathematics of Euclidean geometry (a prototype of axiomatic deductive logic) long appeared sufficient until experimental astronomical observations [27] led to its replacement by more general Riemannian geometry in the early 20th century.

The science of equilibrium thermodynamics offers an exception to how mathematics typically interfaces with physical science. Key underlying regularities (“laws”) of macroscopic thermodynamics were first recognized and formalized by Carnot and others [28] in attempts to understand the scientific principles of steam locomotion in the early 19th century. In the elegant formulation of J. W. Gibbs, the science of thermodynamics came to be firmly based on the *inductive* logic of such laws of experience whose violation has never been observed.

Foregoing sections of this work have suggested how successive conceptual bridges can be employed to further extend the chain of rigorous inductive (thermodynamics-like) inferences from their macroscopic 19th-century origins to the quantal perceptions of the present. This extended “inductive” viewpoint encompasses all materials and natural processes of our observable terrestrial surroundings, down to the level of known atomic, molecular, and supramolecular species.

The coherence and unity of an inductively extended viewpoint is implicitly dependent on the coherence and unity of nature itself, and what can be considered observable (measurable) in nature. This unity suggests that entirely unexpected advances of “pure” mathematics may be initiated when the inductively inferred mathematics of one sub-area is found to be *equivalent* to that which describes the inductive facts of another sub-area. One example is the discovery of quantum mechanics itself, which led to subsequent recognition [29] that the mathematical theory of certain low-order differential equations is *equivalent* to the non-commutative algebra of matrix operations, both of which can be viewed in the more general framework of Hermitian operators in Hilbert space. Another example is summarized in the conclusion, “Thermodynamics *is* geometry” [30], that allows thermodynamic inferences to be drawn in the isomorphic mathematical framework of Riemannian geometry.

## 7. Summary and Conclusions

We have outlined a conceptual framework for extending the inductive logic of macroscopic thermodynamics to the microscopic electronic level of modern computational quantum chemistry, illustrating the underlying computational methodology with a simple application related to chemical mutagenesis. This extension is based on inductive recognition of the infallibility of accurate solutions of the Schrödinger equation in yielding *exact* predictions (within respective computational and experimental error limits) of experimentally measured properties of atomic, molecular, and supramolecular species in all known cases—effectively, an additional *law of nature* since the discovery of the quantum mechanical equations a century ago. The steps of the conceptual ladder connecting macroscopic material changes to molecular-level transformations are the *elementary chemical reactions*, each described by the associated IRC reaction pathway that traverses the non-equilibrium region between equilibrium reactant and product species. Such a stepwise progression through the stationary points of a macroscopic change of state somewhat resembles the finite-time thermodynamics strategy [1] first suggested nearly a half-century ago.

At a more speculative level, we have also suggested that the specific mathematical tools (of thermodynamics, quantum chemistry, statistical mechanics, …) that are found to be effective in dealing with spontaneous natural change may themselves reflect a deeper underlying mathematical unity. Prominent examples include the isomorphism between matrix-algebraic and differential representations that led to recognition of the Hilbert-space underpinnings of quantum mechanics, as well as more recent recognition of the metric geometry underlying equilibrium thermodynamics.

## Data Availability

The original contributions presented in this study are included in the article. Further inquiries can be directed to the author.

## Abbreviations

IRC	Intrinsic Reaction Coordinate
DFT	Density Functional Theoretic
NRT	Natural Resonance Theory
